# Browne on Secretory Influences

**Published:** 1861-06

**Authors:** 


Browne on Secretory Influences.—The latest course of lectures,* by Bernard, was almost exclusively occupied with experimental study of the several secretions which enter the upper portion of the alimentary canal, and in experimentally demonstrating the part directly played in their production by particular nerves.

He satisfactorily demonstrated, in a series of careful experiments, that each of the salivary glands is stimulated to functional activity by a particular nerve; the parotid by a small branch of the facial, the submaxillary by the chorda-tympani, and the sublingual by a branch of the latter.

Having shown experimentally, with uninjured animals, by means of alternate section and irritation of these nerves, that this direct and active relation exists between them and the salivary process, he concludes his illustrations by remarking that "a desideratum still exists," for, notwithstanding section of the chorda-tympani arrests the secretions of the submaxillary and sublingual glands, and galvanic irritation of the peripheric extremity of the divided nerve at once renews the secretions, an "anastomosis is formed between Jacobson's nerve and the chorda-tympani, above the point on which we are (he is) about to divide it; and that to ascertain whether Jacobson's nerve exerts any influence on these secretions, it would be necessary to divide the ninth pair in the immediate vicinity of its cerebral origin—an experiment of such difficulty that no physiologist has hitherto attempted to perform it."†

The suggestion is an anomalous one (since Jacobson's is a sensitive nerve, and Bernard's researches prove that to motor nerves alone are to be assigned direct secretory influences.) It appears to have arisen from a fact noticed by Bernard, which is that "the slender filaments (of the chorda-tympani) connected with the sublingual gland were infinitely less sensible to galvanic irritation than those which fall into the sub-

* Lectures on Experimental Pathology and Operative Physiology. By M. Claude Bernard. Medical Times and Gazette: 1860.

† Medical Times and Gazette, June, 1860.

maxillary plexus."* But Bernard suggests besides, that this difference may be due to difference in structure of those two glands.

However this point may be, his suggestion respecting Jacobson's portion of the glosso-pharyngei (ninth pair) suffices to evince our need of exact and complete knowledge of the physiology of this nerve. The point thus raised by him, if settled, would leave no room for doubt as to the exactness of his extremely valuable present contributions to our physiological knowledge of nervous influence on the salivary glands.

Having, for the benefit of our class, entertained the idea of attempting to attain certainty upon the point thus made by Bernard, we found, on examination, that though a number of experiments are reported by several observers on the ninth pair, some of them were directly contradictory, while others, undertaken to effect a solution of these discrepancies, had led to conclusions equally contradictory in themselves.

This is the case with those of Dr. Jno. Reid, made many years ago.†

His observations, like those of previous observers, fell short of furnishing any satisfactory insight into the physiological relations of this nerve. The alleged experiments of Longet on the nerve are as yet unrepeated by any other observer, so far as I know.

It is plain, from the foregoing, that the attainment of certainty upon the particular pointed out by Bernard, was desirable. However slight its value to medical science, it would leave his brilliant success in fixing upon the particular nerves which enact the part of stimuli to the salivary glands, clear of any anatomical question. As a mere test of the possibilities and limits of operative proceeding on the animal, it deserved a trial; and since no amount of difficulty had ever previously deterred Bernard from attempting the performance of any operation, it seemed decided that he had satisfied himself that its performance without fatal injury to the living animal, was impossible.

Before enumerating the operations performed in our experiments in view of the point in question, we may recall to mind the anatomical course and relations of the ninth pair. In the immediate vicinity of its origin, while within the bones of the

---

* Medical Times and Gazette.

† Todd's Cyclopedia of Anatomy and Physiology, Art. *Glosso-Pharyngeal Nerve.*

skull, it presents two ganglia. Only a part of its fibres pass through the first, but they all are engaged in the second. Within the inner portion of the foramen through which the larger division of the nerve leaves the cranial cavities, it parts with a branch known as Jacobson's nerve, which enters the cavity of the tympanum by a minute bony canal, and divides into several branches. These supply the cavity and carotid plexus of the sympathetic, and anastomose with some filaments of the facial. It is *this* connection alluded to by Bernard. This division takes place very close to its cerebral origin, and no experiment has been made on the nerve above the level of this division.* It is peculiar† that it here receives filaments from the upper or jugular ganglion of the pneumogastric.

The larger division of the nerve, which is all that has been the subject of experiment, after being joined by filaments of the facial, descends to the mucous membrane of the pharynx and the posterior third of the tongue. To these filaments of the facial its motor function is due.

The difficulties mentioned by Bernard, in the way of operating upon the nerve at the point of its origin, we shall merely allude to. They are such in character and number that none but the operative physiologist himself could appreciate them.‡

Some of these arise from the fact, that the operation must be accomplished in the cranial cavity, and the cutting edge of the instrument will, in the most successful operation in the

* Unless those reported of Longet be entitled to be an exception.

† Peculiar, because both are *sensitive* nerves.

‡ An inadequate idea of the *laborious* dissections on the living animal, involved in the repetition of some of his experiments, may be gained from the following statement of Bernard himself, in his description of his discovery of the motor nerves of the parotid gland:—"Our first experiments having been unsuccessful, we modified the operative process." "This last attempt was finally crowned with success after the operation had lasted five hours."

It is, to the student of Bernard, an entertaining circumstance to find Bernard speak of a "difficulty" in any operation. In such an operation as we are about to describe, a peculiar *additional* difficulty exists in the fact, that the operation which has been partially or wholly successful on an animal as one of the preliminary steps, will be utterly useless or misleading as a guide in a repetition of it for similar or additional steps on a second animal, though both may be externally of similar size. This difference depends on the very remarkable variations in different animals of the shape of the skull, its relative thickness, etc. Each skull will differ very remarkably in these and other respects, having a direct bearing upon cranial operations, from every other one.

case, just graze the point of confluence of the lateral and petrosal sinuses.*

And all the difficulties are to be encountered twice in a particular operation, since the nerves are to be divided on both sides.

In the several instances in which the operation was devised and undertaken, most of the various steps were at once accomplished, and subsequently all were successful. The resulting hemorrhage we had feared soon ceased, without any noticeable result. The first operation, upon a medium-sized dog, consisted in drilling through the posterior part of the occipital bone and passing inwards and downwards a suitable instrument, between the dura mater of the lateral border of the cerebellum, and the corresponding portion of the interior of the skull, to the situation of the nerve. In this case the steps of the operation were performed only on one side, and had no injurious result. A noticeable diminution of the sensibility of the parts of the pharynx supplied by the glosso-pharyngei, on the operated side, was the only sign that the nerve had been in whole or part divided.

In the next instance, the operation was varied, for the purpose of ascertaining the possibility of dividing alone either division of the nerve, without the liability to a dangerous result which had been escaped in the first operation.

This was accomplished as before, but now on both sides. When the nerves were thus divided on both sides, the effects upon the animal functions were observed as follows. The operation was ascertained to be successful by these results, and afterwards demonstrated by autopsy.

1st. No difficulty in deglutition, either in eating or drinking, was detected; lapping was performed as usual, nor was it observed that any of the proper motive powers of the parts employed in these functions were in any way altered or impaired.

2d. The ordinary sensibility of the posterior two-thirds of the posterior third of the tongue was found to be lost, and with it the sensibility to taste of this part; while it remained

* This difficulty is constantly encountered by the physiologist in all his operations in the cranial cavity—such as division of the fifth pair. In the dog the sinuses lie so closely together as scarcely to admit the introduction of the smallest *drilling* instrument without opening into them and causing fatal hemorrhage, but if entrance be made into the cavity, and these dangers are escaped, in the next movement in the operation there is the greatest possible liability to other fatal incidents.

intact in the anterior two-thirds. In addition, the sensibility of the upper part of the pharynx, and its tonsilitic and eustachian portions, were lost.

3d. The ducts of the submaxillary and sublingual glands, having been exposed in the manner described by Bernard, the functions of these glands were found to be wholly unimpaired. In the animal on whom this operation had been performed, the application of vinegar to the anterior two-thirds of the tongue always produced a flow of saliva from these glands, through the tubes with which the ducts had been provided.

The ninth pair, therefore, it is ascertained, *exercises no influence on the secretory function of the sublingual or submaxillary glands.*

The filaments derived from that branch of the facial known as the chorda-tympani, alone, therefore, supply the nervous influence on which the secretions of both these glands depend.

Further, we were led to the following conclusions as to the result of section of the glosso-pharyngei.

1st. That since after their section, the functions of deglutition will be performed perfectly well, the impression which leads to this reflex action is due to the gustative branch of the fifth pair, and not to the glosso-pharyngei, as has been invariably alleged.

2d. That while section of the glosso-pharyngei, at the point of their origin, destroys the sensibility of the parts they supply (involving a loss of the power of taste there), the motor power of these parts remains undisturbed. This motor power, therefore, attributed to the pharyngei, must be derived wholly from the fibres of communication received by it from the facial and spinal accessory.

### *Effect of Section of the Facial on Sense of Taste.*

The question of the effects of injury to the facial, on the sense of taste, being still unsettled, we were in proper circumstances, while engaged as above, to observe with reference to it.

Bernard has reported, as the result of his observations, that when the facial nerve is divided or seriously injured, above the point of emergence from the stylo-mastoid foramen, there is a diminished sense of taste on the same side of the tongue. At the same time the general sensibility remains. He considered it certain that this effect is due to the chordatympani, for if the facial be divided *after* the chorda-tympani has left it, no effect is produced on the sense of taste.

In an animal in whom this diminution was effected by division of the facial, the phenomena were observed.

The fact is perplexing, but on attentive consideration was found explicable. The explanation requires, however, an important modification of our theory of taste.

The sense of taste in its peculiar organ differs from the sensibility of other organs in only an *accessory* character, which is established not by any mere modification or alteration of common sensibility, but by the peculiar anatomical structure and action of that organ—a structure which involves *in its peculiarity* the nervous tissue, which, as arranged in other parts, supplies common sensibility only. From this peculiar anatomical constitution of the nervous and other tissue composing the organ arises the *accessory* character, which we distinguish as sense of taste. Besides this, and supplementary to these anatomical conditions, there are certain physiological conditions essential to the perfect performance of the *function* of taste—such as the various and peculiar buccal, facial, and pharyngeal secretions, and associated movements of the various muscular parts. Any modification or disturbance of either of these peculiar anatomical or physiological conditions, will affect *only* the accessory character of the function; but this alteration of the conditions under which we *taste*, will neither disturb nor impair the *common sensibility*, *which is found to be intact in all cases in which this diminution of taste has occurred.*

Any diminution, therefore, of the secretory or motor functions which aid in taste, and which take place through the medium of the facial, and constitute the physiological conditions of the performance of the sense, will be at once manifested by diminishing the perfection of it. A test of this explanation is found in the fact, that the destruction of common sensibility in the tongue involves the loss of all power of taste; and since this is at once accomplished in its anterior two-thirds by division of the lingual branch of the fifth pair, and in its posterior third by division of the glosso-pharyngei, the chorda-tympani being intact, *the latter nerve is not in any sense or degree a nerve of sense to the tongue.* We may consider it settled that its destruction only affects the sense, as a secondary consequence of the withdrawal of its influence from the various secretory and motor functions, upon which the function of taste partly depends.

1. This conclusion agrees substantially with the suggestion offered by Bernard himself, but in addition presents a definite and explicit view of the phenomena of taste. The phenomenon in question so puzzled Stich of Germany (who observed it in a number of cases), that in the absence of a comprehensive and definite explanation, he was driven to attribute it not to the facial, but to fibres derived from the fifth pair. He apparently was not aware that destruction of the fibres of the fifth will occasion, *not* this phenomenon, and not the phenomena of *diminution of taste* with retention of sensibility, but an *extinction* of common sensibility, involving complete *loss* of taste.

In the course of our experiments, we have met with a fact which corroborates this view, viz: that the diminution of taste on division of the tympanitic branch of the facial, does not involve the root of the tongue, a fact which we could make accord with no other explanation. In this part of the tongue, although ordinarily subservient to taste, the anatomical and even physiological conditions, which the accessory character we call taste arise from, are varied. The secretions here are mucoid, and mainly serve the purpose of providing a very slippery coat for the passage of the food through the fauces; while the saliva, freely poured into the anterior part of the mouth, and which especially aids in the gustative function, is mainly mingled with the bolus of food before it reaches the posterior portion. These fluids do not bathe this portion of the mucous membrane, nor exercise their influence on it; nor are the comminuted portions of the food, before they are *amassed* in the bolus, brought into contact with it. In addition to this physiological variation, relating to the two parts, there is the further anatomical difference, that in the posterior part, the secreting structure of the tongue mostly consists of little glands and follicles, providing substances that have but little to do with exciting the gustative process.

In this part, therefore, as we have observed, although its proper sensibility remains, when the glosso-pharyngei are entire, section of the tympanitic branch of the facial has but little if any observable effect; we could not observe *any*.

---

The above is intended merely to present the most abridged form of the effects of section of the glosso-pharyngei, and wholly with reference to the point raised by Bernard.

The experimental evidence, though meagre, is novel. It may, however, find use in the correction of the errors now implicitly adopted by physiologists, which ascribe the reflex function performed in deglutition to the glosso-pharyngei alone.

Its presentation by Bernard, as a point awaiting solution, in intimate connection with his own discoveries, and being valuable as removing the only point of uncertainty respecting them, invested it with an interest and an importance, which the actual results, *since they are known*, will hardly arouse for it in the mind of the reader.—*American Medical Times.*

